# Lysyl Oxidase in Ectopic Cardiovascular Calcification: Role of Oxidative Stress

**DOI:** 10.3390/antiox13050523

**Published:** 2024-04-26

**Authors:** Carme Ballester-Servera, Judith Alonso, Laia Cañes, Paula Vázquez-Sufuentes, Ana B. García-Redondo, Cristina Rodríguez, José Martínez-González

**Affiliations:** 1 Instituto de Investigaciones Biomédicas de Barcelona-Consejo Superior de Investigaciones Científicas (IIBB-CSIC), 08036 Barcelona, Spain; carme.ballester@iibb.csic.es (C.B.-S.); jalonson@santpau.cat (J.A.); paula.vazquez@iibb.csic.es (P.V.-S.); 2 CIBER de Enfermedades Cardiovasculares, Instituto de Salud Carlos III, 28029 Madrid, Spain; ana.garcia@uam.es; 3 Institut de Recerca Sant Pau (IR SANT PAU), 08041 Barcelona, Spain; 4 Instituto de Investigación Hospital La Paz, Universidad Autónoma de Madrid, 28029 Madrid, Spain

**Keywords:** lysyl oxidase, oxidative stress, cardiovascular calcification

## Abstract

Lysyl oxidase (LOX)-mediated extracellular matrix crosslinking modulates calcification in atherosclerosis and aortic valve disease; however, this enzyme also induces oxidative stress. We addressed the contribution of LOX-dependent oxidative stress to cardiovascular calcification. LOX is upregulated in human-calcified atherosclerotic lesions and atheromas from atherosclerosis-challenged LOX transgenic mice (TgLOX^VSMC^) and colocalized with a marker of oxidative stress (8-oxo-deoxyguanosine) in vascular smooth muscle cells (VSMCs). Similarly, in calcific aortic valves, high LOX expression was detected in valvular interstitial cells (VICs) positive for 8-oxo-deoxyguanosine, while LOX and LOXL2 expression correlated with osteogenic markers (SPP1 and RUNX2) and NOX2. In human VICs, mito-TEMPO and TEMPOL attenuated the increase in superoxide anion levels and the mineralization induced by osteogenic media (OM). Likewise, in OM-exposed VICs, β-aminopropionitrile (a LOX inhibitor) ameliorated both oxidative stress and calcification. Gain- and loss-of-function approaches in VICs demonstrated that while LOX silencing negatively modulates oxidative stress and calcification induced by OM, lentiviral LOX overexpression exacerbated oxidative stress and VIC calcification, effects that were prevented by mito-TEMPO, TEMPOL, and β-aminopropionitrile. Our data indicate that LOX-induced oxidative stress participates in the procalcifying effects of LOX activity in ectopic cardiovascular calcification, and highlight the multifaceted role played by LOX isoenzymes in cardiovascular diseases.

## 1. Introduction

Cardiovascular calcification (CVC) is a devastating complication associated with intimal atherosclerosis and valve disease. CVC has become a growing public health issue, with vascular and aortic valve calcifications being prognostic factors for future cardiovascular events [1,2,3]. Regarding the vasculature, microcalcification of the atherosclerotic plaque could increase local mechanical stress and fibrous cap instability, precipitating plaque rupture and causing a thrombotic event. Consistently, the coronary artery calcium score has emerged as a reliable tool to stratify the risk of future cardiovascular morbidity and mortality, independently of traditional risk factors [2,4]. Likewise, in developed countries, calcific aortic valve disease (CAVD) is a highly prevalent cardiovascular disorder affecting up to 25% of the population over 65 years of age [5]. The fibro-calcific remodeling of aortic valve leaflets leads to altered biomechanics, tissue thickening, and compromised motion, characteristic disturbances of aortic valve stenosis that, in the most advanced forms of the disease, could result in systemic afterload, cardiac dysfunction, and heart failure [5]. Despite the increasing progress in our knowledge of CVC etiopathology, surgery and transcatheter aortic valve replacement are the unique therapeutic options for CAVD patients, while no pharmacological tools are available to prevent or slow down CVC.

Initially viewed as a passive degenerative process, CVC is indeed a complex and well-orchestrated phenomenon entailing inflammation, the osteogenic transdifferentiation of resident vascular smooth muscle cells (VSMCs) and valve interstitial cells (VICs), extracellular matrix (ECM) remodeling, and oxidative stress [6]. Certainly, increasing evidence supports the concept that the excessive production of reactive oxygen species (ROS) plays an active role in CVC, fostering the osteochondrogenic differentiation of VSMCs and VICs, while favoring inflammation [7,8,9]. Further, ECM remodeling is a hallmark of CVC and a critical player in biomineralization [10,11]. Recently, we have shown that lysyl oxidase (LOX)-dependent collagen crosslinking drives osteogenic transdifferentiation and modulates mineralization in atherosclerosis and CAVD [12].

The LOX family of enzymes encompasses five members, the archetypal LOX and four LOX-like (LOXL) isoenzymes, which initiate the covalent crosslinking of collagen and elastin fibers, a process responsible for the biomechanical properties of the ECM. Specifically, these enzymes catalyze the synthesis of intra- and intermolecular covalent crosslinks through an oxidative deamination of lysine and hydroxylysine residues of collagen and elastin chains, in a reaction that generates H_2_O_2_ as a by-product [13,14]. Our previous research highlighted the critical contribution of LOX to endothelial homeostasis and its impact on vascular remodeling and stiffness, VSMC proliferation, cardiac hypertrophy, and fibrosis [13,14,15]. Moreover, LOX has been recognized as a source of vascular oxidative stress [16] and a pivotal player in CVC whose expression is upregulated in both CAVD and calcified atherosclerotic lesions as well as in calcifying VSMCs and VICs [12]. However, the potential contribution of LOX to oxidative stress in CVC has not been previously addressed. Here, we provide evidence that the upregulation of LOX in cardiovascular tissues prone to calcification exacerbates local oxidative stress, thereby contributing to cell calcification.

## 2. Materials and Methods

### 2.1. Human Samples

Human aortic valves were collected from CAVD patients who underwent aortic valve replacement at the Hospital de la Santa Creu i Sant Pau (HSCSP; Barcelona, Spain) in accordance with the requirements of the Research Ethics Committee (approval number: 19/267). Aortic valves were categorized based on their macroscopic degree of calcification, as lowly and highly calcified. Lowly calcified (LC) aortic valves were collected from patients with severe aortic valve regurgitation, while those highly calcified (HC) were obtained from patients with severe aortic stenosis. After collection, valves were split into three sections: one was used for the isolation of VICs, a second one was quickly preserved at −80 °C for posterior RNA extraction, and a third piece was processed for immunohistochemical analysis. Human coronary arteries were obtained from patients undergoing heart transplant at the HSCSP (approval number: 19/267). Human femoral arteries were excised from patients undergoing femoropopliteal surgery at the Hospital Clínico Universitario Virgen de la Arrixaca (Murcia, Spain). The studies were approved by their respective Institutional Research Ethics Committee (approval number: 02/10). All research involving human samples adhered to the ethical principles stated by the Declaration of Helsinki of 1975, revised in 2013. Written informed consent was obtained from all patients or their legal representatives.

### 2.2. VIC Isolation and Osteogenic Induction

After collection, valve leaflets were washed in PBS, dissected in small fragments under sterile conditions in a tissue culture hood, and digested by employing 1 mg/mL of collagenase type 2 (LS004176, Worthington Biochemical, Lakewood, NJ, USA), as described [12,17]. Isolated VICs were characterized by immunofluorescence analysis, being positive for α-smooth muscle actin (α-SMA) and vimentin (Figure S1) [12,17]. Mycoplasma contamination was excluded by real-time PCR following the instructions of the Venor^®^GeM qOneStep kit (Minerva Biolabs, GmbH, Berlin, Germany). Cells were plated on gelatin-coated flasks and routinely cultured in DMEM/F-12 (Gibco^TM^, Life Technologies Corporation, New York, USA) supplemented with 20% fetal bovine serum (FBS; Thermo Fisher Scientific, Waltham, MA, USA), 50 ng/mL of insulin, 10 ng/mL of fibroblast growth factor 2, and antibiotics [12,18]. Calcification was induced in low-passage cells by exposure to osteogenic media (OM; high-glucose DMEM supplemented with 5% FBS, 2 mM Na_2_HPO_4_, 50 µg/mL of L-ascorbic acid, and antibiotics), and was analyzed versus cell cultures exposed to control media (CT; high-glucose DMEM supplemented with 5% FBS and antibiotics). LOX activity was pharmacologically inhibited in OM-exposed VICs using β-aminopropionitrile (BAPN; CAS 2079-89-2; 500 µM; Sigma-Aldrich, Merck KGaA, Darmstadt, Germany), as described [10,12]. Cells were treated with BAPN 24 h before osteogenic induction and culture media were subsequently replenished every 3 days. In some approaches, mito-TEMPO (Mito; CAS 1334850-99-5; 25 µM; Sigma-Aldrich) or 4-hydroxy-TEMPO (TEMPOL; CAS 226-96-2; 100 μM; Sigma-Aldrich) was added to osteogenic media, which were replaced every 3 days.

### 2.3. Immunocytochemistry

Primary cultured VICs obtained from human aortic valves were seeded in Permanox^TM^ Chamber Slides (Lab-Tek^TM^, Sigma-Aldrich), fixed in ice-cold 4% paraformaldehyde, permeabilized with 0.4% Triton X-100, and blocked with 1% BSA-PBS. Then, slides were incubated with primary antibodies against vimentin (ab52947, Abcam, Cambridge, UK) and α-SMA (ab5694, Abcam) overnight. After an extensive washing, VICs were exposed to the appropriate biotinylated secondary antibody (Vector Laboratories, Burlingame, CA, USA). Color was developed using 3,3’-diaminobenzidine (DAB; Hoffman-La Roche, Basilea, Switzerland). Cells were counterstained with hematoxylin prior to dehydration and mounting. Negative controls without primary antibody were included.

### 2.4. Analysis of Calcium Deposition in VICs

Calcium deposition in cultured VICs was visualized by staining with 1% alizarin S red (Sigma-Aldrich) and quantified by a blinded operator, as reported [12,17].

### 2.5. Lentiviral Overexpression in VICs

VICs were transduced with recombinant lentivirus to overexpress human LOX using the pLVX/LOX construct previously reported [12]. The empty vector pLVX was used as a control. In brief, 25,000 cells/cm^2^ were seeded into 12- or 48-well plates, transduced for 24 h, and selected with puromycin for 5 days before osteogenic induction, as described [12].

### 2.6. LOX Silencing in VICs

Transient LOX knockdown was carried out in VICs using small interfering RNAs (siRNAs) supplied by the ON-TARGETplus SmartPool System (GE Healthcare Dharmacon, Lafayette, CO, USA) [10,12]. Briefly, 25,000 cells/cm^2^ were transfected into 12- or 48-well plates with a combination of four individual siRNAs (Human *LOX* L-009810-00-0005; GE Healthcare Dharmacon). As a control, the ON-TARGETplus Non-Targeting Control Pool was used (D-001810-10-05; GE Healthcare Dharmacon). Gene knockdown was carried out in VICs using DharmaFECT1 Transfection Reagent (GE Healthcare Dharmacon). Briefly, cells were transfected with 30 nM siRNA and 0.6 μL/cm^2^ of DharmaFECT1 Transfection Reagent. After 8 h, the media were replaced with normal media to allow cell recovery and, after 16 h, cells were cultured under osteogenic conditions, as described [12].

### 2.7. Real-Time PCR

Total RNA was extracted using the TriPure Isolation Reagent (Roche Diagnostics, Mannheim, Germany) following the manufacturer’s instructions. RNA (500 ng) was reverse-transcribed into cDNA using the High-Capacity cDNA Reverse Transcription Kit (Applied Biosystems; Waltham, MA, USA). The quantification of mRNA levels was assessed by real-time PCR using the ABI PRISM 7900 HT sequence detection system (Applied Biosystems) and specific primers and probes (supplied by the Assay-on-Demand system; Applied Biosystems or Integrated DNA Technologies, Coralville, IA, USA) for humans: *LOX* (Hs_00942480_m1), LOX-like 2 (*LOXL2*; Hs_00158757_m1), NADPH oxidase 2 (*NOX2* or *CYBB*; Hs00166163_m1), and osteopontin (*SPP1* or *OPN*; Hs.PT.00261671_m1). mRNA levels were also quantified by SYBR-Green real-time PCR analysis using specific primers targeting the human Runt-related transcription factor 2 (*RUNX2*) (5′-GTCATGGCGGGTAACGATGA-3′ and 5′-AAACTCTTGCCTCGTCCACT-3′). *GAPDH* was used as the endogenous control (Hs.PT.39a.22214836). Relative mRNA levels were determined using the 2^−∆∆Ct^ method.

### 2.8. Animal Handling

Experiments were carried out in transgenic mice that overexpress human LOX specifically to SMC (TgLOX^VSMC^) [12,16]. Non-transgenic C57BL/6J littermates were included as a control group (WT). All experimental procedures were approved by the Institut de Recerca del HSCSP (IRHSCSP) ethical committee in accordance with the Spanish Law (RD53/2013) and the European Directive 2010/63/EU (https://www.boe.es/doue/2010/276/L00033-00079.pdf, accessed on 14 March 2024). Procedures were performed at the Animal Experimental Service in compliance with the principles of 3Rs and ensuring animal welfare.

Atherosclerosis and calcification were induced in 15-week-old male mice by a single tail vein injection of an adeno-associated virus vector (AAV) encoding a gain-of-function mutant of human PCSK9 (AAV-PCSK9^D374Y^; Centro Nacional de Investigaciones Cardiovasculares (CNIC), Madrid, Spain) [12,17,19]. A group of control mice (both TgLOX^VSMC^ and WT) was injected with saline. After 24 h of AAV-PCSK9^D374Y^ administration, mice were fed with a high-fat (HF; 21%) and high-cholesterol (HC; 1.25% added) diet (D12108C, Research Diets, New Brunswick, NJ, USA) for 20 weeks. At the end of this study, vascular samples were dissected, cleaned, and processed for subsequent analysis as described [12,17].

### 2.9. Histology and Immunohistochemistry

Human aortic valves, coronary and femoral arteries, and murine brachiocephalic arteries and aortic arches were fixed in 4% paraformaldehyde/0.1 M PBS (pH 7.4) for 24 h and embedded in paraffin or in OCT and subsequently cryopreserved. Paraffin tissue sections (5 μm) were deparaffinized in two changes of xylene, rehydrated in graded ethanol solutions, and rinsed in distilled water. Antigen retrieval was performed with 10 mM citrate buffer pH 6 or 10 mM Tris/1 mM EDTA pH 9 at 95–99 °C in a water bath for 20 min. Likewise, OCT tissue slides were exposed at 40 °C for 40 min, fixed with cold acetone for 10 min, and extensively washed with PBS. Then, slides were exposed to 3% hydrogen peroxide in methanol for 30 min to block endogenous peroxidase activity. Next, sections were blocked with 10% normal serum and incubated overnight at 4 °C with primary antibodies against vimentin (ab92547, Abcam), LOX (ab31238, Abcam), α-SMA (ab5694, Abcam), RUNX2 (ab236639 (Abcam), and OPN (NB-600-1043, Novus Biologicals, Littleton, CO, USA). Levels of 8-oxo-deoxyguanosine (8-oxo-dG), a biomarker of oxidative stress commonly assessed by immunohistochemistry [20], were also analyzed (ab62623, Abcam). After washing, slides were incubated for 1 h with the appropriate biotinylated secondary antibody (Vector Laboratories, Burlingame, CA, USA) and then exposed to the Vectastain (ABC) avidin–biotin peroxidase complex (Vector Laboratories) for 30 min. Finally, slides were incubated with 3,3’-diaminobenzidine (DAB) and counterstained with hematoxylin before dehydration, clearing, and cover slipping. Calcification was determined in tissue sections by von Kossa staining (Silver plating kit acc to von Kossa, Merck, Darmstadt, Germany) [12,17]. Quantitative analyses were performed by a blinded operator using FIJI ImageJ software (version 1.50i, 2016). Results are expressed as a fold change of positive area vs. fibrous cap area (for brachiocephalic arteries) or lesion area (in aortic arch sections).

### 2.10. ROS Detection

The in situ production of superoxide anions (O_2_^.−^) was assessed with the fluorescent dye dihydroethidium (DHE, Sigma-Aldrich). Briefly, a solution that contained 10 μM DHE and Hoescht 33342 (0.01 mg/mL; Thermo Fischer Scientific) was added to cultured VICs and incubated for 30 min at 37 °C in a light-protected chamber. DHE fluorescence and nuclei were visualized using a confocal microscope (TCS SP5; Leica Microsystems, Deer Park, IL, USA) with an HC PL APO CS20xAir0.70 objective. Images (1024 × 1024 pixels; XY Pixel size: 757.58 nm × 757.58 nm) corresponding to the z-plane with the maximal signal intensity for each captured experimental field were recorded by a blinded operator using a pinhole of 1 A.U. Multiple fields (8–10 fields) containing at least 50 cells were always captured with the same laser conditions. Excitation wavelengths were 405 nm for Hoescht and 561 nm for DHE. Fluorescence emission was captured using sequential scanning. Emission wavelengths were 420–540 nm for Hoescht and 580–750 nm for DHE. Background fluorescence in cell-free areas was equal for all fields analyzed. Fluorescence density was obtained by integrating the DHE fluorescence in each field and normalizing it to the corresponding number of nuclei in the field.

### 2.11. Statistical Analysis

Results are shown as mean ± standard error of the mean (SEM). Significant differences were determined using the unpaired *t*-test, one-way ANOVA, or two-way ANOVA followed by the False Discovery Rate two-stage step-up method from the Benjamini, Krieger, and Yekutieli post hoc test. When data failed the normality test (Shapiro–Wilk test), Mann–Whitney U or Kruskal–Wallis tests were performed or, alternatively, the data distribution was normalized using logarithmic transformation. Correlation analysis of the normal data was assessed using the Pearson test. Data were analyzed with GraphPad Prism version 8.0.2. Differences were considered significant at *p* < 0.05.

## 3. Results

### 3.1. LOX Colocalized with 8-oxo-dG in Human-Calcified Atherosclerotic Lesions

**Figure 1:**
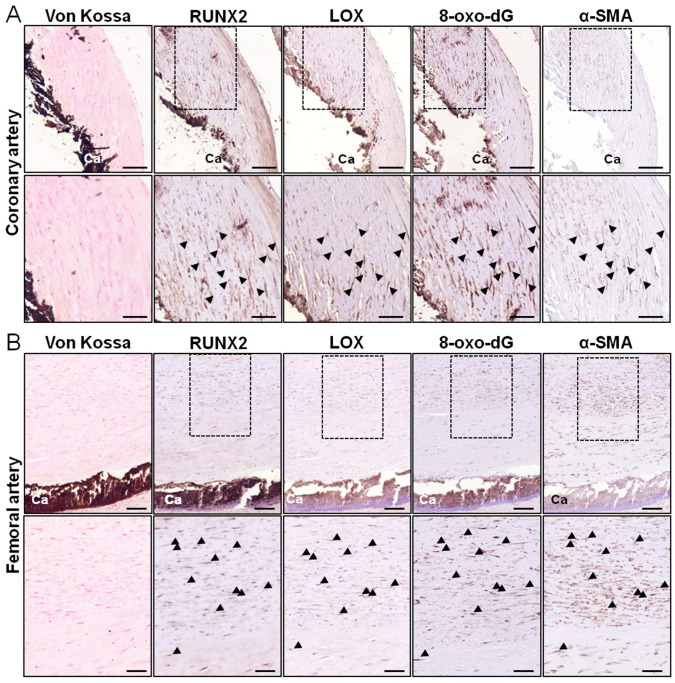
LOX is upregulated and colocalizes with an oxidative stress marker in human-calcified atherosclerotic lesions. Representative images of von Kossa staining and immunohistochemical analysis for RUNX2, LOX, 8-oxo-2′-deosyguanosine (8-oxo-dG), and α-smooth muscle actin (α-SMA) in human-calcified atherosclerotic lesions from coronary (**A**) and femoral (**B**) arteries. The indicated dashed areas are magnified in the lower panels. Black arrowheads indicate positive cells for RUNX2, LOX, 8-oxo-dG, and α-SMA detected in consecutive sections. Bars: 100 µm (**upper** panels) and 50 µm (**lower** panels).

We have previously shown that LOX modulates cell calcification through ECM crosslinking [12]. LOX is also a source of vascular oxidative stress [16]; however, whether LOX activity could contribute to the high ROS production in vascular calcification associated with atherosclerosis has not been previously addressed. Immunohistochemical analysis in serial sections of calcified atheromas from human coronary arteries revealed an intense expression of LOX in VSMCs (α-SMA-positive cells) that express the osteogenic marker RUNX2, close to calcified foci in the intima. Interestingly, in these samples, LOX colocalizes with 8-oxo-dG, a marker of oxidative stress (Figure 1A). A similar LOX immunostaining pattern and colocalization with 8-oxo-dG was found in calcified atherosclerotic lesions from human femoral arteries (Figure 1B). These data suggest that LOX could contribute to the enhanced oxidative stress associated with vascular calcification.

### 3.2. LOX Transgenesis Enhances Oxidative Stress in Atherosclerotic Lesions

**Figure 2:**
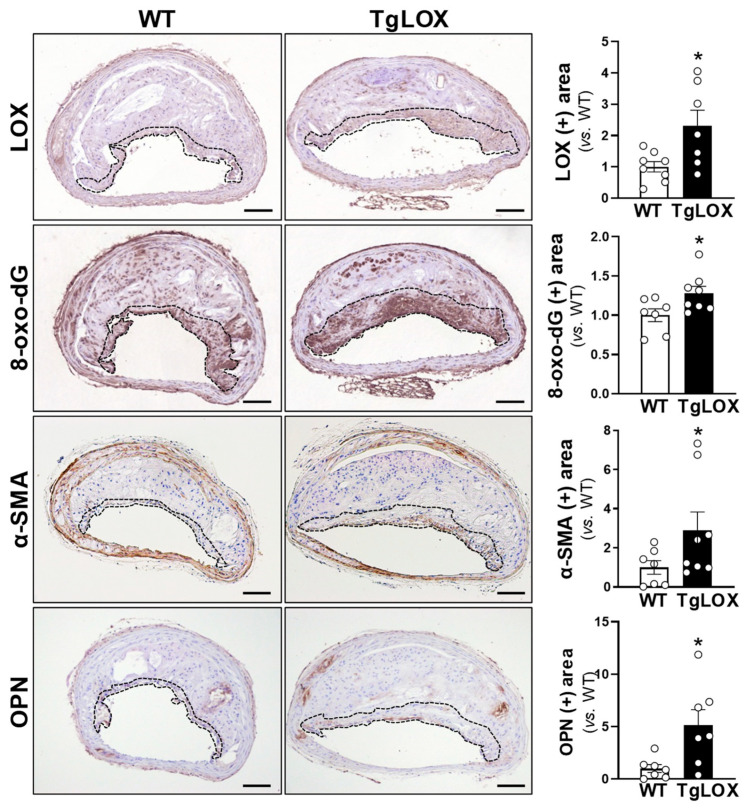
LOX transgenesis enhances oxidative stress and the expression of osteogenic markers in atherosclerotic lesions of the brachiocephalic artery. Wild-type (WT) and transgenic mice that overexpress LOX in vascular smooth muscle cells (TgLOX^VSMC^) were subjected to a single tail vein injection of an adeno-associated virus (AAV) vector encoding for a gain-of-function mutant of human PCSK9 (AAV-PCSK9^D374Y^) combined with a high-fat/high-cholesterol (HF/HC) diet for 20 weeks. Representative images of LOX, 8-oxo-2′-deosyguanosine (8-oxo-dG), α-smooth muscle actin (α-SMA), and osteopontin (OPN) immunostaining in atherosclerotic lesions of the brachiocephalic arteries are shown. Bars: 100 µm. The fibrous cap region is indicated by a dotted line. Bar graphs show the quantitative analysis of immunostaining in the fibrous cap. Results are mean ± SEM. * *p* < 0.05 vs. PCSK9^D374Y^-transduced WT mice (*n* = 7–8).

Next, we addressed whether the exacerbated calcification induced by LOX transgenesis in experimental atherosclerotic lesions [12] could be related to an increase in ROS production. Atherosclerosis and calcification were induced in both transgenic mice that overexpress LOX in VSMCs (TgLOX^VSMC^) and their wild-type littermates (WT) using a single injection of an AAV encoding for PCSK9^D374Y^ combined with an HF/HC diet for 20 weeks, as described [12]. Consistent with that found in calcified human atheromas, in brachiocephalic atherosclerotic lesions, the overexpression of LOX in intimal VSMCs was accompanied by an intense 8-oxo-dG immunostaining, particularly in the fibrous cap (Figure 2). An increased expression of OPN (a maker of osteogenic transdifferentiation) was also detected in TgLOX^VSMC^ lesions (Figure 2), in agreement with the higher susceptibility of the calcification of atherosclerotic plaques from these animals previously reported [12]. Similar results (increased 8-oxo-dG immunostaining in cells overexpressing LOX) were found in the atheromas of the aortic arch from LOX transgenic mice (Figure S2).

### 3.3. LOX and Oxidative Stress in Calcified Human Aortic Valves

**Figure 3:**
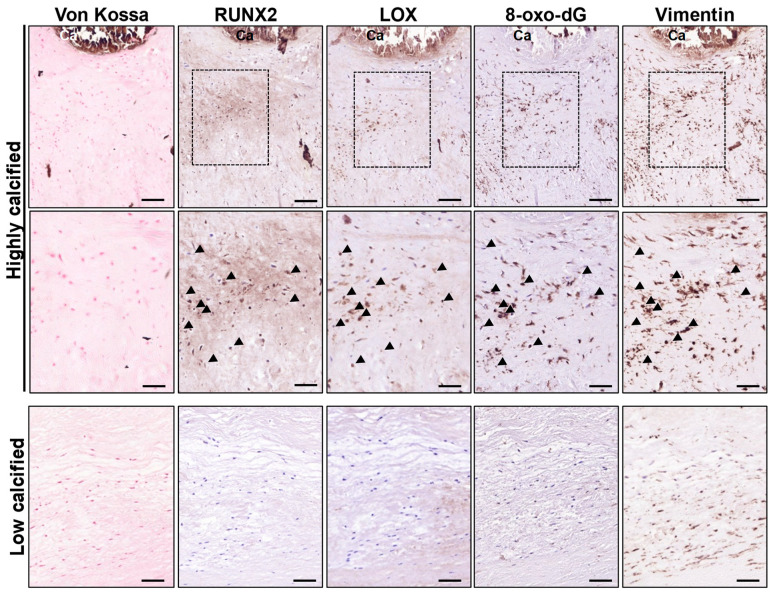
LOX is upregulated and colocalizes with an oxidative stress marker in human-calcified aortic valves. Representative images corresponding to von Kossa staining and immunohistochemical analysis for RUNX2, LOX, 8-oxo-2′-deoxyguanosine (8-oxo-dG), and vimentin in lowly and highly calcified human aortic valves. The indicated dashed areas are magnified in the middle panels. Black arrowheads indicate positive cells for RUNX2, LOX, 8-oxo-dG, and vimentin detected in consecutive sections. Bars: 100 µm (**upper** and **lower** panels) and 50 µm (**middle** panels).

Our previous studies highlighted the role of LOX in the matrix remodeling and osteogenic differentiation of VICs in CAVD [12]. Here, the analysis of consecutive sections from highly calcified human valves shows areas that express RUNX2 and exhibit strong immunostaining for both LOX and 8-oxo-dG in the vicinity of mineralized regions (von Kossa-stained) enriched in VICs (vimentin-positive cells), suggesting that high LOX levels are associated with an excess of oxidative stress (Figure 3). Conversely, LOX and 8-oxo-dG immunostaining were scarce or absent in lowly calcified valves.

**Figure 4:**
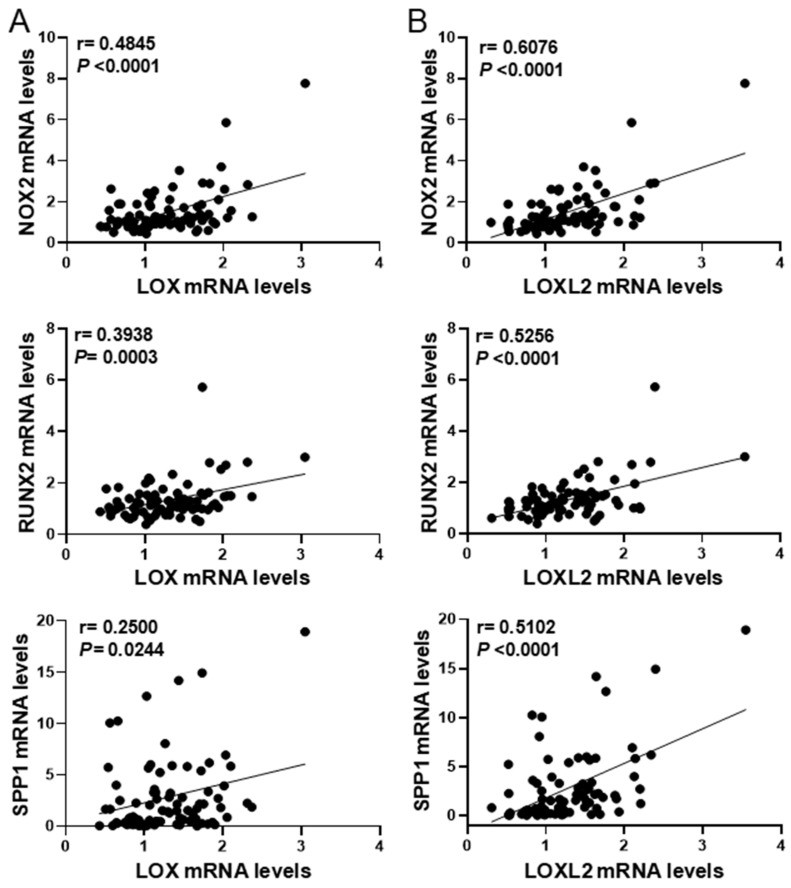
*LOX* and *LOXL2* mRNA levels correlate with the expression of *NOX2* and osteogenic markers in human CAVD. Graphs showing the correlation analysis between *LOX* (**A**) and *LOXL2* (**B**) mRNA levels with those of *NOX2, RUNX2*, and *SPP1* (*n* = 81). The r and *p*-values obtained by the Pearson correlation coefficient test are shown.

In agreement, in aortic valves from our cohort of CAVD patients, *LOX* mRNA levels positively correlated with the expression of the osteogenic markers *RUNX2* and *SPP1* (coding for OPN)*,* as well as *NOX2,* one of the main NADPH oxidases involved in ROS production in CVC [9,21,22,23] (Figure 4A). Similarly, the mRNA levels of *LOXL2*, a member of the LOX family whose expression is also upregulated in highly calcified valves [12], significantly correlated with the levels of *RUNX2*, *SPP1,* and *NOX2* (Figure 4B). These data suggest that LOX activity might play an active role in oxidative stress underlying osteogenic transdifferentiation in CAVD.

### 3.4. Antioxidant Treatment Prevents the Calcification of VIC Cultures Induced by High-Phosphate Media

**Figure 5:**
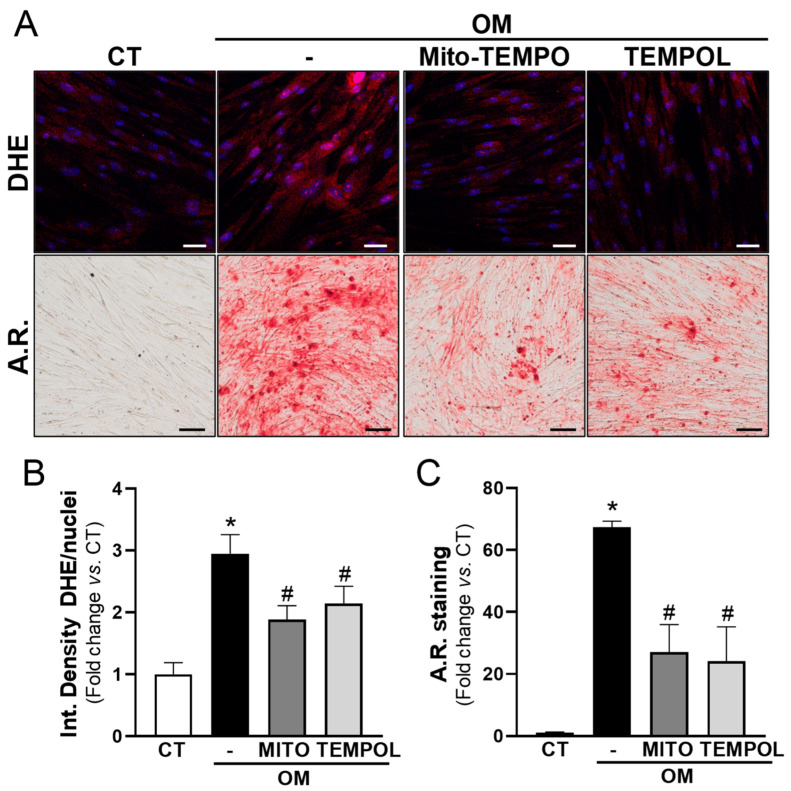
Antioxidants attenuate VIC calcification. Human VICs were maintained in control media (CT, white bars) or cultured under osteogenic conditions (OM, black bars) with or without antioxidant agents (25 μM Mito-TEMPO [MITO; dark grey] or 100 μM TEMPOL [light grey]). Superoxide anion production and mineralization were assessed by dihydroethidium (DHE) and Alizarin Red (A.R.) staining, respectively. (**A**) Representative images of DHE (**upper** panels, red signal) and A.R. staining (**lower** panels) are shown. Nuclei in upper panel are stained with Hoescht (in blue) Bars: 50 µm in upper panels and 100 µm in lower panels. (**B**,**C**) The histograms show the result of the quantitative analysis of DHE (**B**; *n* = 8) and A.R. staining (**C**; *n* = 9). Data are mean ± SEM. *p* < 0.05: * vs. cells cultured in CT media; # vs. cells cultured in OM in the absence of antioxidant agents.

The results described above encouraged us to investigate whether the calcification of human VICs is highly dependent on ROS. OM increased the generation of superoxide anions (detected by DHE staining) and the mineralization of VICs (Figure 5). Interestingly, the antioxidant agents mito-TEMPO (25 µM) and TEMPOL (100 μM) partially prevented the increase in superoxide anions and strongly reduced calcium deposition (Figure 5).

### 3.5. LOX Increases Oxidative Stress and Mineralization of VICs

**Figure 6:**
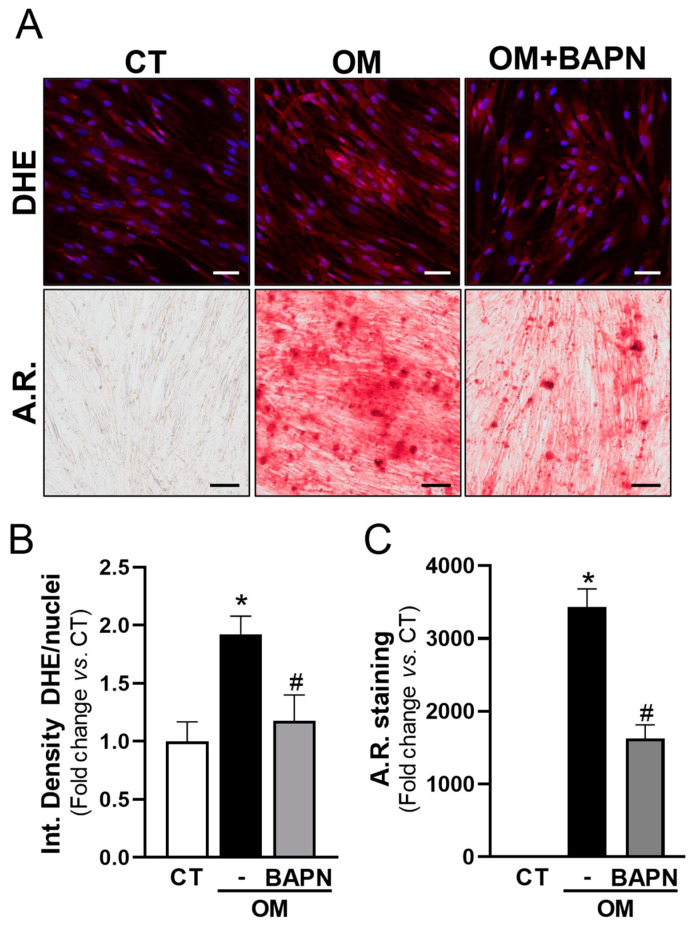
Inhibition of LOX activity reduces ROS production and mineralization in calcifying VICs. Human VICs were cultured in control (CT, white bars) or osteogenic media (OM) in the presence (grey bars) or absence (black bars) of 0.5 mM β-aminopropionitrile (BAPN). Superoxide anion production and mineralization were assessed by dihydroethidium (DHE) and Alizarin Red (A.R.) stains, respectively. (**A**) Representative images of DHE (**upper** panels; in red) and A.R. staining (**lower** panels) are shown. Nuclei in upper panel are stained with Hoescht (in blue). Bars: 50 µm (**upper** panels) and 100 µm (**lower** panels). (**B**,**C**) Quantitative analysis corresponding to DHE (**B**; *n* = 8) and A.R. staining (**C**; *n* = 9). Data are mean ± SEM. *p* < 0.05: * vs. cells cultured in CT media; # vs. cells cultured in OM in the absence of BAPN.

In view of these data and because LOX is a source of oxidative stress in different scenarios [15,16,24,25], we aimed to assess whether LOX could significantly increase ROS levels in calcifying VICs. LOX activity was inhibited in VICs exposed to OM using BAPN. Notably, BAPN was as effective as antioxidant compounds in reducing both superoxide anion levels and mineralization (Figure 6).

**Figure 7:**
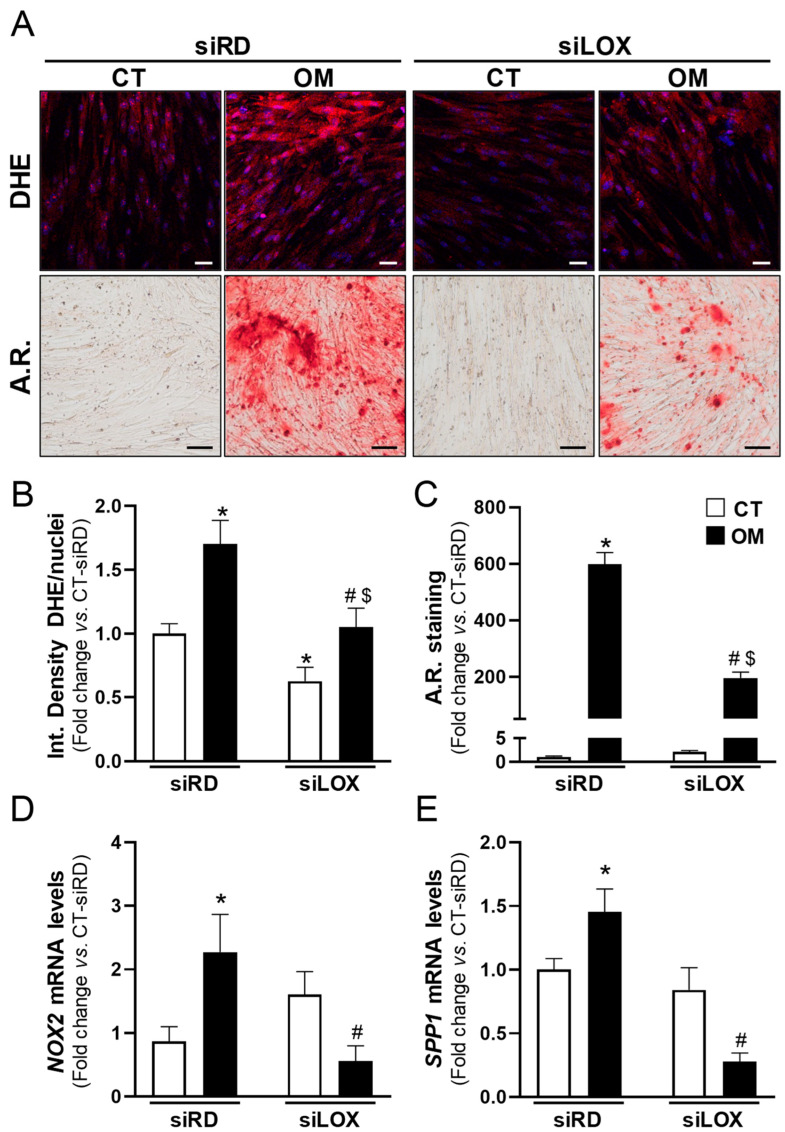
LOX knockdown attenuates oxidative stress and mineralization in calcifying VICs. Human VICs were transfected with an siRNA against LOX (siLOX) or a Random siRNA (siRandom, siRD) and exposed to control (CT, white bars) or osteogenic conditions (OM, black bars). Superoxide anion production and mineralization were assessed by dihydroethidium (DHE) and Alizarin Red (A.R.) staining, respectively. (**A**) Representative images of DHE (**upper** panels; in red) and A.R. staining (**lower** panels) are shown. Nuclei in upper panel are stained with Hoescht (in blue). Bars: 50 µm in upper panels and 100 µm in lower panels. (**B**,**C**) Bar graphs show the quantitative analysis of DHE (**B**; *n* = 9) and A.R. staining (**C**; *n* = 9). (**D**,**E**) mRNA levels of NADPH oxidase 2 (*NOX2*) and *SPP1* (coding for osteopontin) assessed by real-time PCR (*n* = 5–6). Data are mean ± SEM. *p* < 0.05: * vs. siRD-transfected cells cultured in CT media; #, vs. siRD-transfected cells exposed to OM; $, vs. siLOX-transfected cells under CT conditions.

**Figure 8:**
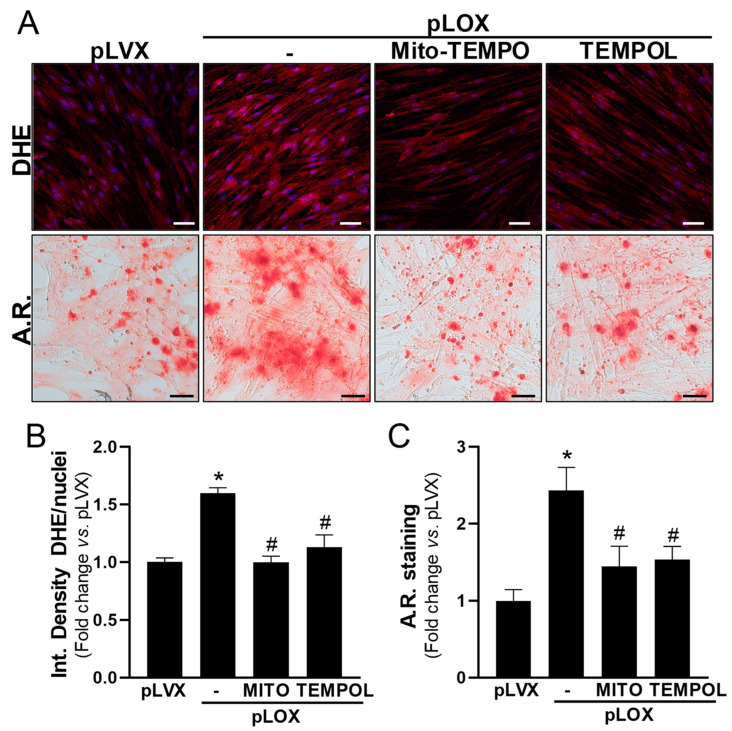
Antioxidant treatment in VICs overexpressing LOX decreases both ROS production and calcification. Human VICs were transduced with pLVX (empty vector) or pLVX-LOX (pLOX) lentivirus and were cultured under osteogenic conditions in the presence or absence of antioxidants agents (25 µM Mito-TEMPO [MITO] or 100 µM TEMPOL). Superoxide anion production and mineralization were assessed by dihydroethidium (DHE) and Alizarin Red (A.R.) staining, respectively. (**A**) Representative images of DHE (**upper** panels; in red) and A.R. (**lower** panels) staining are shown. Nuclei in upper panel are stained with Hoescht (in blue). Bars: 50 µm. (**B**,**C**) Quantitative analysis of DHE staining (**B**; *n* = 8) and A.R.-positive area (**C**; *n* = 9). Data are mean ± SEM. *p* < 0.05: * vs. pLVX-transduced cells; # vs. pLOX-transduced cells in the absence of antioxidant agents.

**Figure 9:**
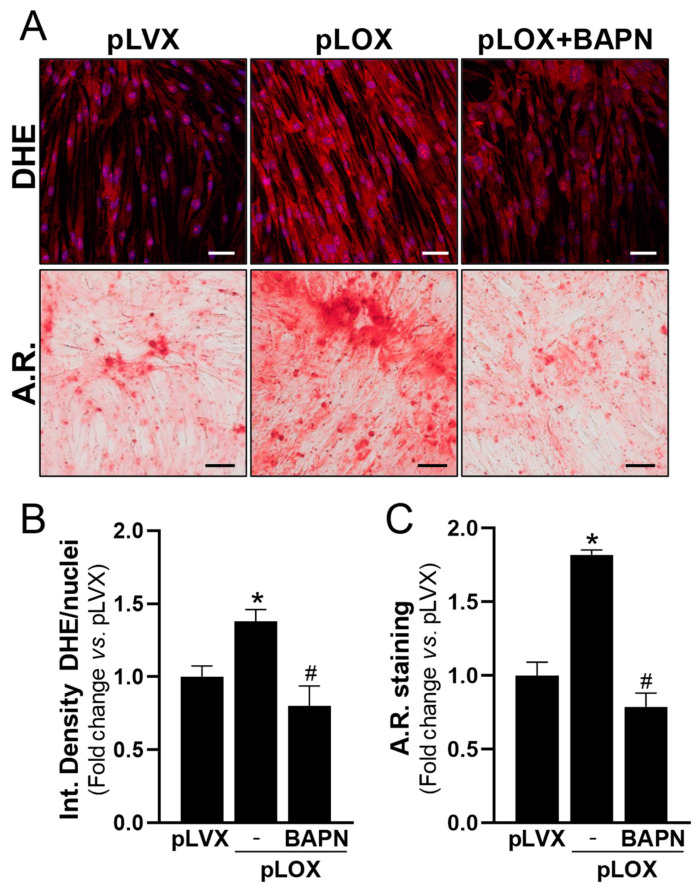
Lentiviral overexpression of LOX in VICs increases both ROS production and calcification. Human VICs were transduced with pLVX (empty vector) or pLVX-LOX (pLOX) lentivirus and were cultured under osteogenic conditions in the presence or absence of 0.5 mM β-aminopropionitrile (BAPN). Superoxide anion production and mineralization were assessed by dihydroethidium (DHE) and Alizarin Red (A.R.) staining, respectively. (**A**) Representative images of DHE (**upper** panels: in red) and A.R. (**lower** panels) staining are shown. Nuclei in upper panel are stained with Hoescht (in blue). Bars: 50 µm in upper panels and 100 µm in lower panels. (**B**,**C**) Quantitative analysis of DHE (**B**; *n* = 10) and A.R. staining (**C**; *n* = 9). Data are mean ± SEM. *p* < 0.05: * vs. pLVX-transduced cells; # vs. pLOX-transduced cells in the absence of BAPN.

In agreement, in cells exposed to OM, the specific knockdown of LOX (Figure S3A) attenuated the increased levels of ROS and the exacerbated calcification of cell cultures, as well as the higher mRNA levels of the pro-oxidant enzyme *NOX2* and the osteogenic marker osteopontin (encoded by *SPP1*) (Figure 7). Conversely, the overexpression of LOX in VICs by lentiviral transduction (Figure S3B) increased both the generation of superoxide anions and the mineralization in response to OM (Figure 8 and Figure 9). In the presence of either antioxidant compounds (Figure 8) or BAPN (Figure 9), ROS levels were similar to those of pLVX-transduced cells and calcium deposition was significantly attenuated. Altogether, these data indicate that LOX-derived ROS contributes to the enhanced VIC mineralization induced by this enzyme.

## 4. Discussion

The molecular mechanisms that trigger and drive CVC, a disorder which significantly impacts on cardiovascular risk, are not fully understood. The ECM-modifying enzyme LOX is involved in the pathophysiological mechanisms underlying cardiovascular diseases [13,14,15], and we have recently described that LOX-mediated collagen crosslinking plays a critical role in osteogenic cell differentiation and in the calcification of both atherosclerotic lesions and aortic valves [10,12,26]. In the present study, by analyzing human and animal samples and applying different pharmacological and gain- and loss-of function experimental approaches, we conclude that LOX-induced oxidative stress seems to contribute to the procalcifying effects of this enzyme, which highlights the multifaceted role played by this family of enzymes in the pathophysiology of cardiovascular diseases.

The analysis of calcified atherosclerotic lesions from two different human vascular beds showed that the increased expression of LOX in VSMCs associates with high 8-oxo-dG immunostaining. 8-oxo-dG is one of the main forms of the free radical-induced oxidative modification of nuclear and mitochondrial DNA, a stable oxidative modified form of DNA extensively used as a biomarker for oxidative stress in several disorders including cardiovascular diseases [27,28,29]. Indeed, enhanced immunoreactivity against 8-oxo-dG has been previously reported in human atheromas from carotid endarterectomy specimens [30]. We have extended this observation to human-calcified atheromas from coronary and femoral arteries, in which 8-oxo-dG was detected in the vicinity of calcified foci, colocalizing with LOX, thus suggesting that LOX activity might contribute to the excess of oxidative stress observed in vascular calcification. The relationship between LOX and ROS production in atherosclerotic vascular calcification was also found in a mouse model that overexpresses LOX in VSMCs (TgLOX^VSMC^) [12,26]. Indeed, consistent with findings in human atheromas, atherosclerotic lesions from these transgenic mice prone to intimal calcification [12,26] exhibited enhanced 8-oxo-dG in VSMCs colocalizing with LOX.

Oxidative stress is increased in calcifying tissues and actively contributes to the potentiation of other mechanisms involved in CVC [9,31,32,33,34]. Multiple sources of ROS have been associated with the pathogenesis of vascular calcification including the NADPH oxidase system, xanthine oxidases, and uncoupled nitric oxide synthases, among others. LOX has consistently been related to oxidative stress in different pathological scenarios [15,16,24,25]; however, whether LOX-derived ROS play a role in vascular calcification has not been previously addressed. Certainly, H_2_O_2_ is generated as a by-product of LOX activity [13,14]. In fact, some biological responses attributed to LOX rely on this key molecule for redox signaling and oxidative stress such as the LOX-dependent regulation of VSMC chemotaxis, vascular stiffness, and cancer cell migration [16,35,36]. In the context of our study, this is of particular interest, because H_2_O_2_ promotes VSMC osteoblastic transdifferentiation through the AKT-mediated induction of RUNX2 [37], an essential transcription factor for osteochondrogenic differentiation and calcification. Accordingly, our previous data revealed that, in VSMCs, the overexpression of LOX increases RUNX2 protein levels and subsequently promotes matrix mineralization [10], while high RUNX2 expression is detected in calcified atherosclerotic lesions from TgLOX^VSMC^ mice [12].

Early studies by Liberman et al. support that ROS and particularly H_2_O_2_ promote aortic valve calcification. Using DHE and DCF-DA staining, these authors showed that oxidative stress increases in CAVD, mainly around calcifying foci [22]. This was corroborated by other authors that found high superoxide and H_2_O_2_ near the calcified regions of valves from CAVD patients [38,39]. Using 8-oxo-dG immunostaining, we also detected increased oxidative stress in the vicinity of focal mineral deposits in highly calcified human valves, and, as in calcified atherosclerotic lesions, LOX was mainly expressed by 8-oxo-dG-positive VICs, suggesting the contribution of this enzyme to oxidative stress in CAVD. Moreover, in these samples, the expression of LOX and LOXL2 significantly correlated with NOX2. This NADPH oxidase, which is commonly upregulated in human and experimental CAVD in regions with high levels of H_2_O_2_ and OPN close to mineralized nuclei [22], is actively involved in CAVD [21]. Our previous studies support a reciprocal crosstalk between LOX-derived H_2_O_2_ and NADPH oxidase [16], a mechanism that could explain the correlation found between the expression of LOX and LOXL2 with NOX2 in CAVD samples. As expected, in these samples, the mRNA levels of both LOX isoenzymes correlated with osteoblastic markers, in agreement with our previous report showing that LOX activity modulates the osteogenic transdifferentiation and mineralization of VICs [12]. Obviously, LOXL2, whose expression is also upregulated in highly calcified valves, contributes in generating H_2_O_2_ in calcified tissues, although due to its lower expression in relation to LOX, it seems to play a secondary role in CAVD [12].

Our studies in cultured VICs further strengthen the concept that oxidative stress is an active player in ectopic CVC. Indeed, antioxidants such as TEMPOL and mito-TEMPO attenuated the increased oxidative stress and the subsequent deposition of calcium triggered by the osteogenic media. Multiple studies have shown a protective effect of antioxidant molecules, antioxidant enzymes, or mimetics of these enzymes, against experimental calcification [39,40,41,42,43,44,45,46], pointing to oxidative stress as a key mechanism in CVC [9,47,48], and supporting the rationale of therapeutic strategies targeting ROS for managing CVC [47]. However, ROS are pleiotropic signaling agents essential for many cellular processes [9,49]. This could explain the negative results of clinical trials addressing the therapeutic use of high doses of non-selective antioxidants [50,51]. Therefore, efforts should be focused on specific elements participating in the generation of supraphysiological levels of ROS. In this context, the specific targeting of the LOX enzyme in calcified tissues could be valuable, because it not only merely participates in the exacerbated oxidative stress underlying CVC, but it also actively contributes to osteogenic transdifferentiation and matrix mineralization [10,12]. Our gain- and loss-of-function experimental approaches in VICs cultured under osteogenic conditions showed that LOX inhibition, by pharmacological or gene knockdown strategies, abolished both the enhanced oxidative stress and the deposition of calcium. By contrast, the lentiviral overexpression of LOX triggered the opposite response, aggravating ROS production and matrix mineralization, effects that were prevented by both antioxidant compounds and BAPN. Therefore, ectopic CVC may be one of the pathologies that could benefit from the therapeutic potential of selectively targeting LOX family members [14,52].

## 5. Conclusions

Our study shows that LOX critically contributes to oxidative stress in cardiovascular calcification and holds promise for targeting LOX-derived ROS as an interesting strategy for therapeutic intervention.

A limitation of this study is the quantification of oxidative stress and matrix mineralization by image analysis. The determination of 2-hydroxyethidium by HPLC and the *o*-cresolphthalein method could be alternative approaches to assess such parameters.

## Data Availability

The original contributions presented in the study are included in the article/Supplementary Material, and further inquiries can be directed to the corresponding authors.

## Supplementary Materials

The following supporting information can be downloaded at https://www.mdpi.com/article/10.3390/antiox13050523/s1, Figure S1. Immunohistochemical staining for VIC markers. Figure S2. LOX transgenesis enhances oxidative stress and the expression of osteogenic markers in atherosclerotic lesions of the aortic arch. Figure S3. Efficient LOX knockdown and lentiviral LOX overexpression in human valvular interstitial cells (VICs).
