# Supplementary material for: Lysyl Oxidase in Ectopic Cardiovascular Calcification: Role of Oxidative Stress
Source: Antioxidants (Basel). 2024 Apr 26;13(5):523. doi: 10.3390/antiox13050523 (PMC11118817; doi:10.3390/antiox13050523)
Supplement: Supplementary file 1 [file antioxidants-13-00523-s001.zip › antioxidants-2942031-supplementary.pdf]

SUPPLEMENTAL MATERIAL

Lysyl oxidase in ectopic cardiovascular calcification: role of oxidative stress

Carme Ballester-Servera,[1,2,3] Judith Alonso,[1,2,3] Laia Cañes,[1,3] Paula Vázquez-Sufuentes,[1,3]

Ana B. García-Redondo [2,4], Cristina Rodríguez,[2,3†] José Martínez-González[1,2,3†]

[1]Instituto de Investigaciones Biomédicas de Barcelona-Consejo Superior de Investigaciones Científicas (IIBB-CSIC), Barcelona, Spain.
[2]CIBER de Enfermedades Cardiovasculares, Instituto de Salud Carlos III, Madrid, Spain.
[3] Institut de Recerca Sant Pau (IR SANT PAU), Barcelona, España;.
[4]Instituto de Investigación Hospital La Paz, Universidad Autónoma de Madrid, 28029 Madrid, Spain.

† These authors contributed equally to this work

Address for correspondence: Correspondence should be addressed to J.M.-G. IIBB, Rosselló, 161, 08036 Barcelona, Spain (email: jose.martinez@iibb.csic.es; phone: +34 93 5565896) or C.R. IRHSCSP, C/Antoni Mª Claret, 08025 Barcelona, Spain (email: crodriguezs@santpau.cat; phone: +34 93 5565897).

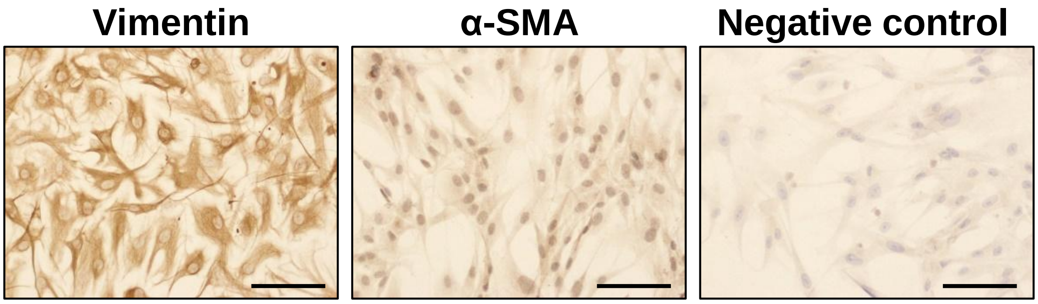

Figure S1. Immunohistochemical staining for VICs markers. Representative images showing immunostainings for vimentin (left panel) and α smooth muscle cell (α-SMA; middle panel) in primary cultures of valvular intersticial cells (VICs) from human aortic valves. Negative control in which primary antibody was omitted is shown (right panel). Bars: 100 µm.

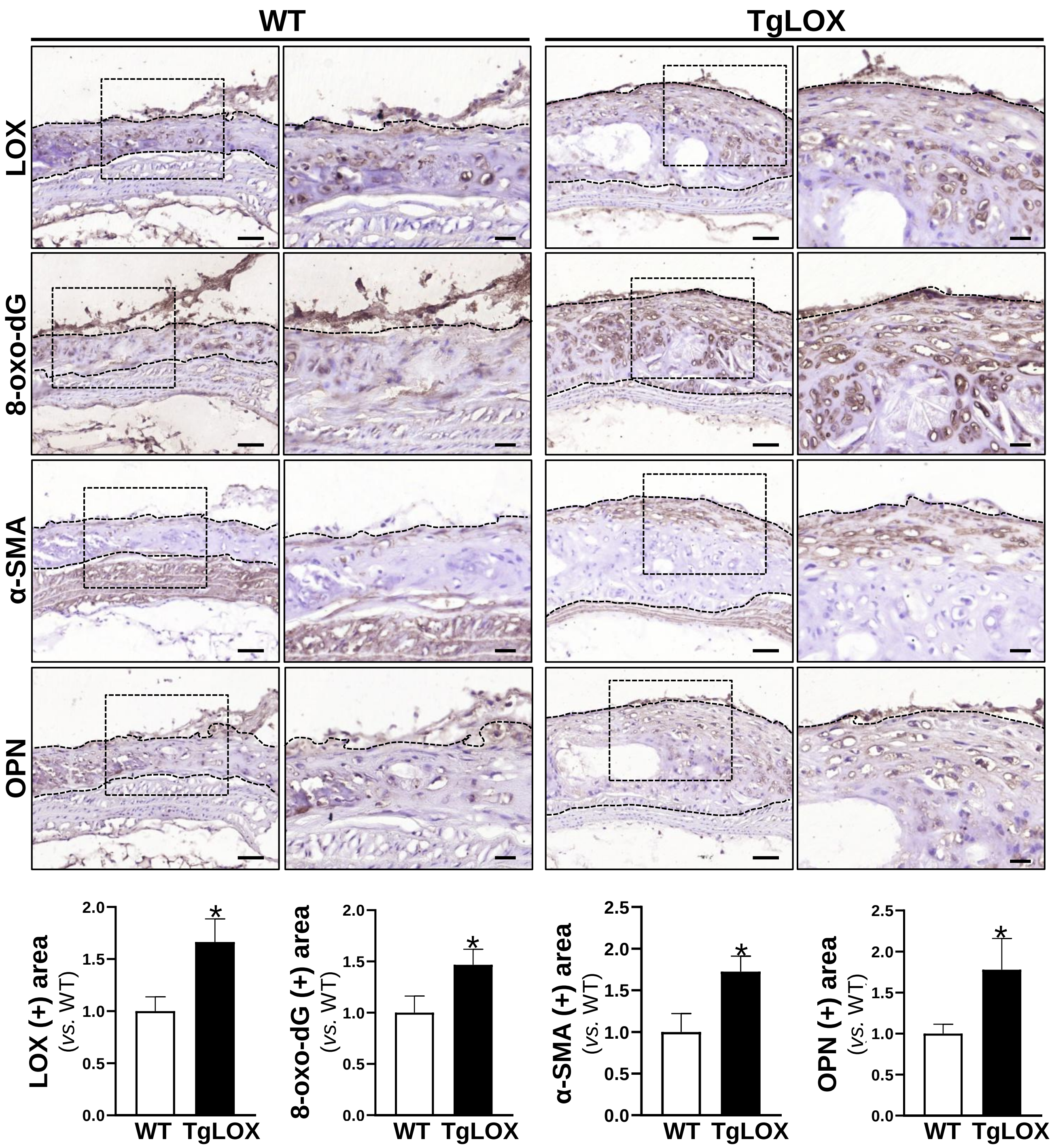

Figure S2. LOX transgenesis enhances oxidative stress and the expression of osteogenic markers in atherosclerotic lesions of the aortic arch. Wil-type (WT) and transgenic mice that overexpress LOX in vascular smooth muscle cells (TgLOX^{VSMC}) were subjected to a single tail vein injection of adeno-associated virus (AAV) vector encoding for a gain-of-function mutated form of human PCSK9 (AAV-PCSK9^{D374Y}) combined with a high fat/high cholesterol (HF/HC) diet during 20 weeks. Immunostaining for LOX, 8-oxo-2'-deosyguanosine (8-oxo-dG), α-smooth muscle actin (α-SMA) and osteopontin (OPN) in the aortic arch are shown. Lesion areas are indicated with dotted lines and boxed areas are magnified in right panels. Bars: 50 µm (left panels) and 20 µm (magnified panels). Bar graphs show the quantitative analysis of immunostainings of intimal lesions indicated by a dotted line. Results are mean±SEM. *$P<0.05$ *vs.* PCSK9^{D374Y}-transduced WT mice (n= 5-7).

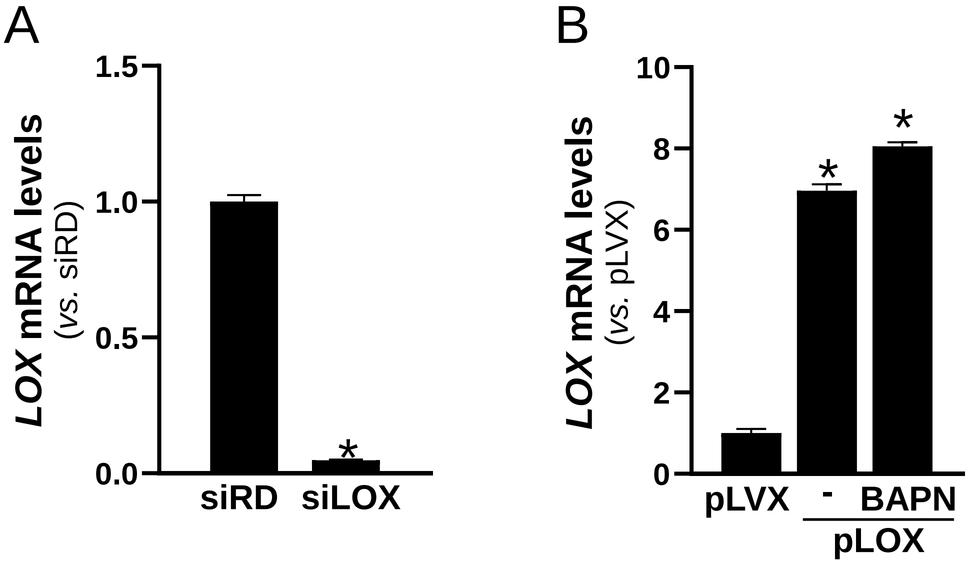

Figure S3. Efficient LOX knockdown and lentiviral LOX overexpression in human valvular interstitial cells (VICs). (A) Human VICs were transfected with a siRNA against LOX (siLOX) or a Random siRNA (siRD). LOX mRNA levels were assessed by real-time PCR. Data are mean±SEM (n= 6). *P*<0.001: **vs*. siRD-transfected cells. (B) Human VICs were transduced with pLVX (empty vector) or pLVX-LOX (pLOX) lentivirus in the presence or absence of 0.5 mM BAPN (β-aminopropionitrile; inhibitor of LOX activity). LOX over-expression was verified by real-time PCR. Data are mean±SEM (n= 4). *P*< 0.001: **vs*. pLVX.
